# Combining Biocompatible and Biodegradable Scaffolds and Cold Atmospheric Plasma for Chronic Wound Regeneration

**DOI:** 10.3390/ijms22179199

**Published:** 2021-08-25

**Authors:** Steffen Emmert, Sven Pantermehl, Aenne Foth, Janine Waletzko-Hellwig, Georg Hellwig, Rainer Bader, Sabine Illner, Niels Grabow, Sander Bekeschus, Klaus-Dieter Weltmann, Ole Jung, Lars Boeckmann

**Affiliations:** 1 Clinic and Policlinic for Dermatology and Venereology, University Medical Center Rostock, 18057 Rostock, Germany; sven.pantermehl@med.uni-rostock.de (S.P.); aenne.foth@med.uni-rostock.de (A.F.); ole.jung@med.uni-rostock.de (O.J.); 2 Department of Oral, Maxillofacial and Plastic Surgery, University Medical Center Rostock, 18057 Rostock, Germany; janine.waletzko-hellwig@med.uni-rostock.de; 3 Clinic and Policlinic for Orthopedics, University Medical Center Rostock, 18057 Rostock, Germany; georg.hellwig@med.uni-rostock.de (G.H.); rainer.bader@med.uni-rostock.de (R.B.); 4 Institute for Biomedical Engineering, University Medical Center Rostock, 18119 Rostock, Germany; sabine.illner@med.uni-rostock.de (S.I.); niels.grabow@med.uni-rostock.de (N.G.); 5 ZIK Plasmatis, Leibniz Institute for Plasma Science and Technology (INP), 17489 Greifswald, Germany; sander.bekeschus@inp-greifswald.de (S.B.); weltmann@inp-greifswald.de (K.-D.W.)

**Keywords:** skin regeneration, wound healing, plasma medicine, disinfection, blood flow enhancement, electrospinning, natural and synthetic nanofibers, asymmetric membranes

## Abstract

Skin regeneration is a quite complex process. Epidermal differentiation alone takes about 30 days and is highly regulated. Wounds, especially chronic wounds, affect 2% to 3% of the elderly population and comprise a heterogeneous group of diseases. The prevailing reasons to develop skin wounds include venous and/or arterial circulatory disorders, diabetes, or constant pressure to the skin (decubitus). The hallmarks of modern wound treatment include debridement of dead tissue, disinfection, wound dressings that keep the wound moist but still allow air exchange, and compression bandages. Despite all these efforts there is still a huge treatment resistance and wounds will not heal. This calls for new and more efficient treatment options in combination with novel biocompatible skin scaffolds. Cold atmospheric pressure plasma (CAP) is such an innovative addition to the treatment armamentarium. In one CAP application, antimicrobial effects, wound acidification, enhanced microcirculations and cell stimulation can be achieved. It is evident that CAP treatment, in combination with novel bioengineered, biocompatible and biodegradable electrospun scaffolds, has the potential of fostering wound healing by promoting remodeling and epithelialization along such temporarily applied skin replacement scaffolds.

## 1. Introduction

In acute wounds, for example, surgery or trauma, a primary wound closure via sutures is desired. Chronic wounds, however, affect a large part of the elderly population, rising to 2%–3% in 80 year-olds [1]. In such non-healing wounds, the process of wound closure is disturbed or retarded at least in one of the three phases of wound healing. Therapeutic strategies aim for wound disinfection and stimulating applications or procedures to activate the healing process [2,3]. Despite such modern wound dressings and procedures, wounds often persist over months and even years. Thus, new, additively acting and wound healing promoting interventions are urgently needed that may markedly reduce treatment time and, thus, related treatment costs. Cold atmospheric plasma (CAP) with its multimodal mechanisms of action definitely constitutes such an innovative intervention [4,5]. CAP reduces the bacterial load on wounds and re-initiates the stagnated healing process [2,6]. The plasma cocktail contains free electrons and ions, UV irradiation, electrical fields, and reactive oxygen and nitrogen gas species (RONS) that mediate different biological effects towards tissue regeneration within up to 90 seconds of treatment [7]. In addition, a body of research has emerged with new tissue engineering developments that combine new fabrication processes such as electrospinning with innovative biocompatible and even degradable wound dressing materials, including nanofibers, hydrocolloids, and other natural or synthetic polymers serving as soft tissue scaffolds. The interdisciplinary field of tissue engineering and the regenerative medical field has been rapidly growing during the last two decades. The aim of tissue engineering is the replacement of lost tissue and restore tissue function. Multifunctional 3D bioscaffolds are developed to stimulate the regrowth of tissue. Bioactive scaffolds can be enriched with living cells of different origins, bioactive molecules or enzymes to not only provide mechanical strength and guidance for autologous tissue regrowth but also restore functional tissue properties [8,9]. Target tissues for artificial engineering include bone, cartilage, muscle, nerves, cardiovascular tissue and skin. Cardiac tissue engineering appears to be especially challenging, as special characteristics of the tissue surface have to be achieved [10]. Another interesting target tissue is bone tissue. Here, high mechanical strength combined with osteointegration properties and biodegradability are a prerequisite for biomimetic bone tissue engineering [11]. Scaffolds, especially artificially constructed ones, may also be coated to modify surface properties (e.g., hydrophobic) and enhance attachment to the remaining tissue to be restored. Jaroszewicz et al. recently described the production of calcium phosphate coatings of scaffolds [12]. In addition to non-natural scaffold materials, scaffolds from natural products are also under investigation. One example is chitinous materials as they are available in abundance and are eco-friendly. Large scale isolation can be performed from marine sponges and seafood or fungal biomass. The advantages as well as challenges of chitinous materials include the isolation process and cryopreservation [13,14]. Machalowski et al. recently described moulting cuticles of spiders as an alternative source of naturally occurring chitin [15]. Finally, to highlight human-derived scaffolds, decellularized tissues have been utilized in regenerative/tissue engineering medical applications. Gilbert et al. summarized different decellularization methods [16].

Of special interest for skin replacement are asymmetric membranes that consist of two layers that resemble the functions of the epidermis and dermis. Such electrospun scaffolds enhance porosity, the absorption of moisture, exchange of oxygen, and antimicrobial properties as well as outer protection and barrier function [17,18,19,20,21,22,23]. Another innovative way to produce such a bilayered skin scaffold is constituted by high hydrostatic pressure (HHP). With the HHP method, human skin tissue may be devitalized gently thereby maintaining its biomechanical properties in the sense of devitalized allogenic skin tissue scaffolds [24]. This may even be extended by bioprinting stem cells or platelet-rich plasma or disinfectants or drugs or other wound healing enhancers into the scaffolds via 3D printing technologies [25].

## 2. Skin Regeneration

### 2.1. Causes of Chronic Skin Wounds

Skin wounds comprise a great variety of skin destructions with a loss of skin tissue. Many different classifications exist to distinguish wounds by depth of tissue loss, wound structure, or form. Within this context, skin ulcerations are loss of skin tissue comprising all three skin layers (epidermis, dermis, subcutaneous fat tissue). Such ulcers per definitionem always heal with scarring. Another often applied wound classification is based on the persistence of the skin ulcer: acute versus chronic. Such a skin defect—if more severe—may also include subcutaneous structures like muscles, tendons, or even bones and is regarded as chronic if no signs of healing are detectable over a period of 8 weeks or longer [26]. Besides burn wounds, chronic wounds are generally the result of malnutrition of skin cells that results from reduced blood flow in the papillary capillaries. Per diffusionem, epidermal keratinocytes are nourished from there. This applies to constant tissue pressure wounds (decubitus), venous wounds (tailback of capillary blood), diabetic wounds (microangiopathy of capillary skin arterioles), as well as peripheral arterial circulatory diseases. Chronic wounds in addition lead to a considerable reduction of the quality of life due to pain, odor, or secretion. Moreover, the disruption of the epidermal barrier is an open door for the entry of pathogens that may lead to life-threatening septic infections. With new knowledge on pathophysiology followed by the developments of new therapeutic healing technologies more effective wound management plans can be developed [27].

**Figure 1:**
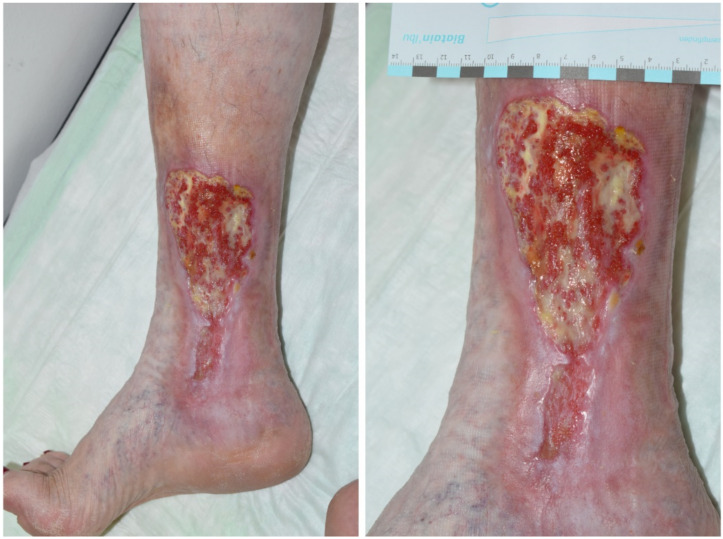
Chronic venous leg ulcer typically located at the inner malleolus due to varicosis of the vena saphena magna. Note all three stages of chronic venous insufficiency according to Widmer: Corona phlebectatica at the bottom of the ulcer (Widmer stage I), liposclerosis with brownish hyperpigmentation at the top of the ulcer, atrophie blanché and purpura jaune d’ocre (Widmer stage II) as well as the ulcer itself (Widmer stage III). Left: overview; right: detailed view.

Varicosis resulting in chronic venous leg ulcers (Figure 1) vastly prevails among other causes of leg ulcers (80%) [28]. The prevalence is estimated to be up to 1% of an elderly population, for example, in Germany, up to 800,000 people are affected [29]. With increasing age there is a gradient in the prevalence from 60 to 80 years [30]. In general, it is reported that it takes approximately 24 weeks until a leg ulcer heals. Unfortunately, the recurrence of ulcerations is high and up to 71%, and 15% of all ulcers may never heal [31,32]. The prevalence of leg ulcers in people with diabetes is even higher and reaches up to 6%. Amputation of the foot or a toe in the case of diabetes as the cause of an ulcer may reach up to 0.8% of cases [3].

These high prevalence rates in the elderly are also paralleled by the high costs of wound treatment. This poses a high socio-economic burden for the public health system in general. It is estimated that 1–2 percent of the budget for public health care is spent on ulcer treatment, adding up to 900 million Euros [28,29]. These estimations are exclusive of further indirect costs of chronic ulcers. Sick leave (ulcers cause 1.2% of all sick leave days) and early retirement costs have to be taken into account, too, as well as hospitalization as in-patients [3]. These cost considerations also contribute to the need for new skin allografts, scaffolds and treatment modalities to enhance wound healing and treatment efficacy.

### 2.2. Stages of Wound Healing

The healing of skin defects that often include defects in subcutaneous tissues is a complex orchestrated process [3]. Three phases of wound healing can be roughly discerned: (i) Cleaning phase with coagulation and inflammation; (ii) granulation phase with matrix synthesis, angiogenesis, fibrosis, and tissue proliferation; (iii) re-epithelialization with tissue remodeling and scar formation [33,34,35].

Usually, a tissue defect includes vessel disruption and bleeding. This is initially desired to expel foreign bodies, but bleeding has to come to an end after a while. To this end, platelets initiate the process of coagulation and begin a cascade of hemostatic reactions. Clotting factors are released as well as different kinds of cytokines that mediate inflammatory processes to further foster wound cleaning [36]. In this process, the platelet-derived growth factor (PDGF) appears to be essential, working in concert with the transforming growth factors A1 (TGF-A1) and 2 (TGF-2) and attracting muscle cells, fibroblasts, macrophages, and neutrophilic leukocytes into the wound area. Interestingly, reactive oxygen species (ROS) are released by leukocytes and macrophages to combat germs and bacteria within this cleaning reaction. Other cytokines involved include transforming growth factor-beta (TGF-β) that activates macrophages among other cells. Elastases and matrix metalloproteinases (MMPs) eliminate destroyed tissue together with amines secreted from mast cells to lay the grounds for new vessel and tissue formation. Dolor, rubor, and calor comprise typical clinical symptoms during this inflammation phase, gradually resolving some days after wounding [37,38]. To bring the inflammatory phase to an end, anti-inflammatory cytokines (interleukin-1, TGF-A1), as well as bioactive lipids (resolvons, lipoxons, prostagaglandines), come into play [39,40].

Granulation comprises the second phase of wound healing. Here, cells of different types, including fibroblasts and endothelial cells, proliferate and release a plethora of growth factors. Such factors include the epidermal growth factor (EGF), transforming growth factor-beta (TGF-β), or vascular endothelial growth factor (VEGF). Interestingly, increased lactate levels, a reduced pH level, and reduced partial oxygen pressure pave the way for a favorable wound healing milieu [37,41,42]. That an acidic environment fosters and promotes wound healing is well known [43]. It is also well established that the factors necessary for granulation also support re-epithelialization and the final closure of the skin.

Finally, skin epithelialization can be considered the third phase of wound healing. The restoration of epidermis and re-epithelialization and restored blood supply in the dermis appear to be essential for keratinocyte proliferation and migration [3,34]. Several other cell types are also active in the epithelialization phase; these include macrophages and immune cells. As in the second phase of wound healing, certain cell types release a special set of cytokines, chemokines and integrins. Metalloproteinases are also involved in the remodeling of the second and outer most skin layers. Pastar et al. also nicely reviewed the role of keratinocytes and re-epithelialization [44].

### 2.3. Modern Wound Therapy

Despite a nearly endless variety of wound dressings and wound therapeutic options [45], efficient wound therapy still remains an urgent need, especially for treating chronic wounds [46]. It is evident that efficient treatments depend on the wound condition, for example, location and depth, bacterial colonization, extent of wound exudation, and the phase of healing that is hampered. It is notable that, besides compression therapy and phlebo-/arterial surgery, all other wound treatments are merely symptomatic and non-causative.

Usually, the first step of wound treatment is the mechanical removal of fibrin layers, bacterial biofilm and dead and necrotic tissue. This is usually achieved by surgical debridement. However, other debridement technologies exist, such as pulse-lavage systems or ultrasonic wave cleaning [26,47]. Enzymatic ointments or even treatments with maggots can also be applied for wound cleaning. Washing with physiological saline solution is often performed or with disinfectants such as octenidin solution. Then, the wound dressing is applied, which has to comply with multiple functions. This includes keeping the wound moist but yet allowing air and oxygen exchange. Yet, wound exudate needs to be stored. The materials need to be biocompatible and non-irritative for the skin as well as hypoallergenic and bio-compostable after use. Active dressings harbor antibiotics, drugs, or other substances such as silver to prevent multipathogenic overload or growth factors to initiate proliferation [44,48,49].

To this end, complex requirements exist for the development of new topical wound therapies. On the one hand, sufficient biocompatibility has to be ensured for new materials. On the other hand, materials should support tissue regeneration, be biologically active and antiseptic. Achieving a dose window of antimicrobial and anti-biofilm activity with simultaneous tissue compatibility is a great challenge in the field [49].

## 3. Cold Atmospheric Plasma (CAP)

### 3.1. What Is Cold Atmospheric Plasma (CAP)

Physical plasma is regarded as the fourth state of matter after solids, liquids, and gases. Plasmas can be produced by adding energy to an otherwise neutral gas. Originally, plasma medicine was introduced by Nikola Tesla in the 1890s [50]. Physical plasmas may be divided into thermal (hot) and non-thermal (cold) plasmas. For decades, thermal plasmas have been used for industrial applications. They are used, for example, for surface treatment or for cutting different kinds of materials. Thermal plasmas have also been applied in medicine for more than 20 years. They provide the most prevalent procedure for endoscopic tissue coagulation [51] and help with hemostasis during surgical interventions. Thermal plasmas are also used for the devitalization and ablation of tissues, for example, to remove tumors from the bladder [52], to treat wards, actinic keratosis, hemangiomas, [53] or for the treatment of enlarged nasal concha [54]. As thermal plasmas can reach very high temperatures, they are not suited for gentle application to living cells, tissues or temperature sensitive medical instruments. For such biomedical applications, only biocompatible non-thermal plasmas are suitable. Cold atmospheric pressure plasmas possess a mixture of diverse biologically active agents. These include various reactive oxygen species including ozone, various reactive nitrogen species, as well as charged atoms, ions, electrons, UV radiation, visible light and electromagnetic fields. These components work in concert and impose a number of different biological effects on the skin. Plasma application may have anti-inflammatory, anti-itch, pain-relieving, antibacterial, tissue stimulating, and capillary blood flow stimulating effects, but also pro-apoptotic, tumor cell killing effects [55,56].

**Figure 2:**
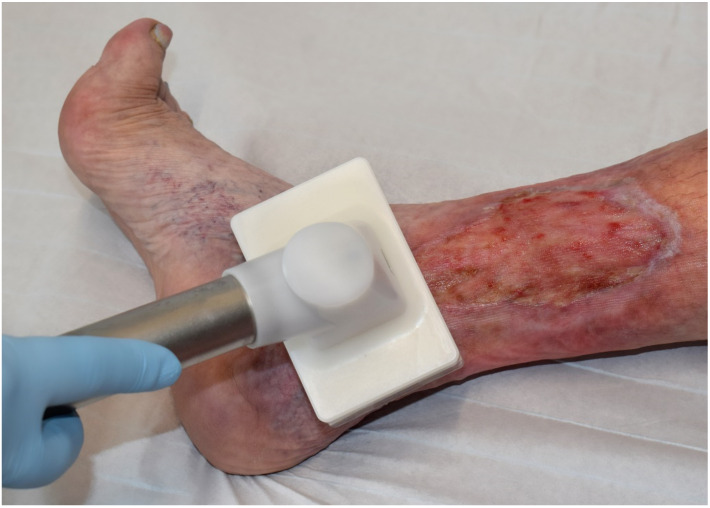
Application of the DBD plasma device PlasmaDerm^®^ (CINOGY GmbH, Duderstadt, Germany) to a venous ulcer on the lower leg. The advantage of the DBD device is coverage of a greater treatment area. The flexible sponge electrode is ideal for flat wounds.

In general, two ways of generating cold atmospheric pressure plasmas can be distinguished: First, dielectric barrier discharge (DBD) based devices (direct plasma discharge) and second, jet plasma devices (indirect plasma discharge) [57]. DBD devices consist generally of two laminar electrodes, of which one electrode is shielded by an insulating (dielectric) layer. This results in a very low conduction current despite a high voltage and the generated plasma is thermally barely warming up. An example for such a DBD plasma source provides the PlasmaDerm device (CINOGY GmbH, Duderstadt, Germany; Figure 2). This device allows the treatment of larger areas of several square centimeters and the treatment is independent of additional gas supply.

**Figure 3:**
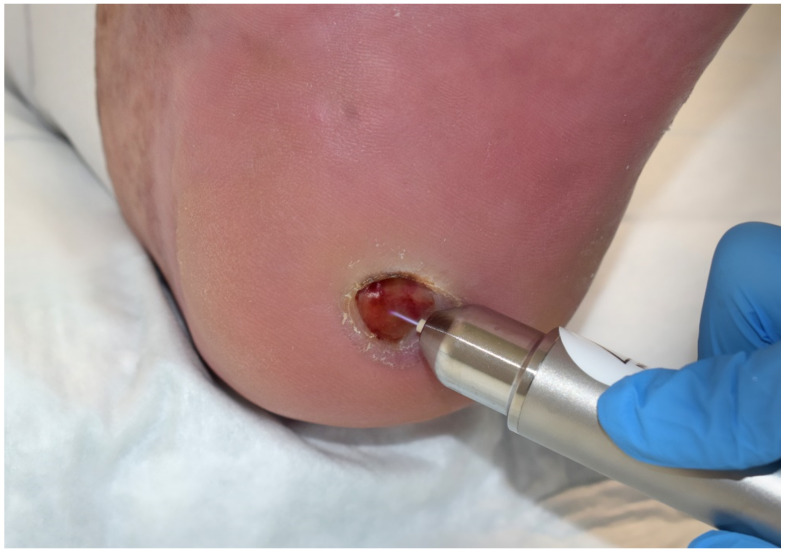
Application of the plasma jet device kINPen^®^ MED (neoplas med GmbH, Greifswald, Germany) to a diabetic pressure ulcer on the foot. The advantage of the jet device is plasma transduction into deeper wound areas and into wound lashes.

In jet plasma sources, the required electrodes are arranged in or on a nozzle. A feed gas is guided through the nozzle and is ionized by applying a high voltage to the electrodes. With the flow of the feed gas, the plasma is blown out of the nozzle. With such devices, several different gases such as argon, helium, or air can be used. This allows, within certain limits, a variability of the composition of the plasma components. Furthermore, plasmas from jet plasma devices may be applied into the smallest caps and pores. A well-established example for such a cold atmospheric plasma jet provides the kINPen MED (neoplas med GmbH, Greifswald, Germany; Figure 3), which uses argon as feed gas.

Based on several case reports and clinical studies the two mentioned devices PlasmaDerm and kINPen MED could have been CE-certified in 2013 as medical products (class 2a) for the treatment of chronic wounds and microbial induced diseases of the skin and hence were approved for application in the clinic.

The biologic effects of cold plasma are considered to be mainly due to reactive species which act on cells and may react with proteins and lipids. Reactive oxygen species, such as hydroxyl radicals, superoxide and singlet oxygen, or reactive nitrogen species, such as nitrogen oxides and peroxynitrite, seem to be most important in mediating efficacy [6,58].

### 3.2. Cold Atmospheric Plasma (CAP) and Wound Healing

A number of case reports and small clinical studies first addressed clinical safety and the reduction of bacterial load as primary endpoints and thereby paved the road for CAP into clinical practice [59,60,61,62,63,64]. Some of these studies also looked at wound healing as a secondary endpoint or retrospectively.

Meanwhile, the efficacy of plasma application for improved ulcer healing was confirmed and validated in at least three comprehensive prospective, randomized, and controlled studies. The first involved 50 patients with pressure induced ulcers [65]. The assessment of wound size reduction revealed markedly enhanced wound healing after plasma treatment compared to standard care. To date, the largest and best elaborated prospective multicenter study focused on feet ulcers in diabetics using the kINPen MED device. Here, in total, 65 ulcers and 45 patients were treated [6]. Half of the wounds received plasma treatment eight times and the other half was mock treated (argon gas flow without plasma ignition). All wounds in addition received modern standard wound care. The authors found that in the plasma treated group wounds healed quicker and the ulcer size reduction was faster. Astonishingly, neither a pronounced reduction in bacterial wound load nor in wound site infections was detectable in the plasma treated group. This may be a hint that ulcer size reduction may not be too dependent on bacterial infections. Another third trial reported comparable results to the aforementioned study [66]. Again, all 44 diabetic patients received modern standard care and 22 patients received plasma treatment in addition. However, here, plasma not only resulted in faster wound healing but also in an immediate reduction of bacterial load, followed, however, by quick bacterial recolonization. Finally, a most recent prospective study showed that plasma ulcer treatment once a week was not inferior to treatments three times per week [67].

A systematic review of CAP applications in dermatology and especially of wound healing analyzing 166 studies also comes to the conclusion that CAP treatment acts beneficially. The study emphasizes that CAP can act in concert with other drugs and optimize percutaneous drug delivery [68].

## 4. Bioengineering of Biocompatible and Biodegradable Scaffolds

The use of xenografts, allografts and autografts are widely established methods to cover skin defects permanently [69]. However, especially autologous skin grafts are not available in abundance, and their application necessitates the establishment of additional wounds in the patient [70,71]. To overcome such limitations, artificial skin scaffolds/wound dressings are applied to wounds temporarily until the physiological healing process has been completed [72,73]. As mentioned above, wound dressings ideally combine different functions in one three-dimensional structure to keep the wound moist and allow air exchange. This would be best achieved by a structure mimicking the epidermal/dermal bilayer structure of the outer skin [74]. The core structure of such a scaffold should mimic the dermis and extracellular matrix (ECM). This is ideally accomplished by a 3D network of biocompatible and biodegradable nanofibers with high porosity that allows cells to adhere, proliferate and differentiate [75,76,77]. Sufficient mechanical strength is another prerequisite of such a scaffold [17]. A so-called asymmetric membrane would in addition contain an outer layer with rather low porosity, that is, small pores and hydrophobic properties [78]. PHEMA-based hydrogels may be one option for building up dual porosity scaffolds. Macroporous hydrogels based on cross-linked poly(2-hydroxyethyl methacrylate) (pHEMA) have been assessed in recent years for their swelling and mechanical properties as well as their ability to foster cell adhesion and proliferation with a special focus on stem cells. PHEMA hydrogel properties were assessed with light and electron microscopy, laser scanning confocal microscopy as well as micro-CT analysis and oscillatory shear measurement [79,80,81].

The core material of such scaffolds can be natural, semi-synthetic, or fully synthetic [82]. The advantages of natural nanofibrous polymers are usually good biocompatibility with no hindrance of cell adhesion, proliferation, and differentiation, as such materials share similarities with dermal ECM structures [83]. Bioengineers use silk [84], collagen or gelatine [85,86], hyaluronic acid [87], or chitosan [78] and chitin [88,89]. Insufficient mechanical stress tolerance may constitute the major drawback of natural nanofibers [90]. The advantages of synthetic nanofibers are their availability in abundance at usually lower costs compared to natural materials, and their stiffness and elasticity can be designed as needed. This also applies to biodegradability. Polylactic acid (PLA), poly-D,L-lactide-co-glycolic acid (PLGA), polyvinyl alcohol (PVA), or polycaprolactone (PCL) are widely used synthetic polymers [17,20]. To utilize the advantages of both materials and to overcome the limitations of each, bioengineering evaluates mixtures of natural and synthetic materials as nanofibrous scaffolds [91].

Several technologies can be applied for the production of monolayered EMC-like nanofibrous scaffolds, including self-assembly, phase separation, extraction, drawing, or electrospinning. As other technologies are tedious, not easily up-scalable for mass production or in need of post-synthetic processing steps it appears that electrospinning is most feasible for wound scaffold preparation that has been optimized during recent years [92]. For bilayer, asymmetric membrane production scCO_2_-assisted phase inversion, wet or dry/wet phase inversion, and again, electrospinning, have been utilized [93]. Electrospun biosystems made of nylon 6 and laccase [94] or horseradish peroxidase immobilized onto electrospun fibers [95] may be utilized for environmental protection, for example, for the industrial removal of dyes or phenolic pollutants from wastewater.

**Figure 4:**
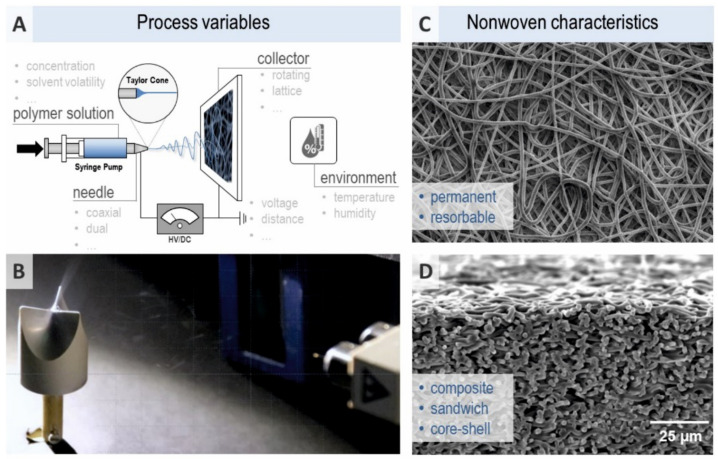
(**A**) Schematic of the electrospinning (ES) principle, (**B**) Macrograph of dual ES for 3D heart valve replacement, (**C**) SEM image, demonstrating the highly porous surface morphology of an ES nonwoven sample, (**D**) Cross-sectional SEM image, demonstrating the compact bulk architecture and pronounced interfibrous integrity of the ES sample.

Electrospinning works with electrostatic fields to produce nanofibers from a polymeric solution of a size between 50 to 1000 nm. The electrospinning setup includes a syringe with a capillary needle and syringe pump for the polymeric solution, a spinneret charged with high voltage and low current and a grounded metallic collector. An electric field is applied between the needle and the collector. The solution at the tip of the syringe forms a so-called Taylor cone due to the highly charged surface (conical shape). Electrostatic and Columbic forces stretch and dehydrate the polymer solution ejected from the needle towards the collector, resulting in a thin and dry fiber on the collector. With the needle tip shape (e.g., co-axial, multi-jet, multifluidic co-axial) and collector shape the organization of the fibers can be orchestrated (Figure 4). Ambient conditions such as atmosphere pressure, air velocity, or humidity as well as operating conditions such as applied voltage, temperature, flow rate of the solution, the diameter of the syringe tip, and the tip to collector distance constitutes the electrospinning setup parameters [96]. Such parameters, together with viscosity, surface tension, or conductivity of the solution, determine the mechanical strength and porosity of the membrane. Applying rotating collectors allows for special topographic and spatial arrangements of the scaffold [97] regarding a more homogeneous fiber orientation and size distribution.

The safe and effective application of micro- and nanofibrous nonwovens as part of biomedical products requires a high stability of the electrospinning process parameters. In particular, process validation as part of product approval and continuous quality assurance is focusing on reliable high-throughput tools for fiber diameter measurement. Here, recent advances in automated scanning electron microscopy (SEM)-based imaging may provide substantial benefits regarding accuracy, processing speed and statistical power [98].

Pamu et al. [17] have recently performed a systematic review and nicely summarize the results of applying one material for scaffold formation. PLA [99], collagen [100], a combination of chitosan, PVA, and zinc oxide [101], a blend of curdlan and PVA [102] were studied among others in the treatment of chronic wounds in diabetic patients. The scaffolds induced macrophage activation, secretion of cytokines (inflammatory healing phase), reduced cyclooxygenase-2, induced nitric oxygen synthesis, influenced the expression of various growth factors like VEGF, TGF-β, or EGF and in in-vivo examinations accelerated wound healing as well as re-epithelialization. Similarly, bilayered, asymmetric scaffolds showed enhanced cytocompatibility, fluid take-up, cell infiltration, and regeneration using blends of chitosan-PCL and hyaluronic acid [78] or chitosan and PDLA [103]. In-vivo application in a mouse wound model revealed accelerated healing using a β-glucan acetate and β-glucan butyrate scaffold composition [104].

**Figure 5:**
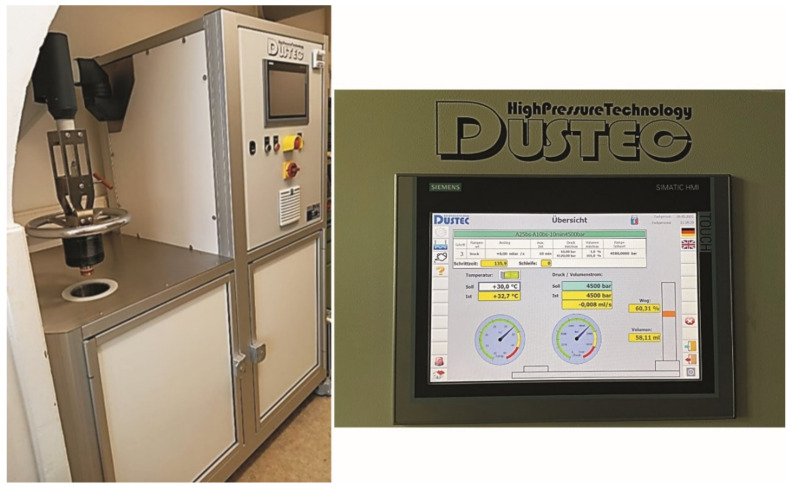
Pressure testing system (HighPressureTechnology Dustec, Wismar, Germany), with a sample volume of 200 mL. High hydrostatic pressures up to 600 MPa can be applied. Temperatures between 0 °C and 40 °C can be set constantly during HHP treatment.

Besides the electrospinning of artificial skin scaffolds, another yet to be explored approach to produce human bilayered skin scaffolds is high pressure biotechnology [105]. High hydrostatic pressure (HHP) treatment can devitalize cells while preserving the structure of the skin in the sense of a matrix structure maintaining biomechanical properties (Figure 5). The idea would be to devitalize human skin (epidermis/dermis obtained from lately deceased) to produce a highly biocompatible yet cell and pathogen-free human skin scaffold without the need for nanofabrication. That this is, in principle, feasible has been shown for bone replacements [106] and cartilage repair [107]. In a pilot animal study, Hiemer et al. [107] devitalized osteochondral tissue applying HHP. They used a rabbit model to fix osteochondral defects. The authors found that in the knee joint their HHP scaffolds could replace osteochondral defects. Moreover, HHP scaffolds guided osteochondral cells towards the defective site. Thus, HHP-treated skin could also have the potential to act as functional scaffold for wound healing.

Finally, nanofabricated skin scaffolds or HHP-treated skin could be augmented by substances, cells, enzymes, or drugs to further foster wound healing. To this end, essential oils [108], doxycycline [109] and other antibiotics [110], or growth factors like PDGF [111] or VEGF [112] have been added to bilayered scaffolds showing additive healing effects. Thus, scaffolds can be utilized as drug delivery systems [113,114,115,116]. It is noteworthy that CAP treatment would be an ideal combinatorial treatment with such wound scaffolds as CAP is not only stimulating wound healing, angiogenesis, acidification, and cell proliferation but also acts as skin penetration enhancer. Lademann et al. [117] showed that nanoencapsulated drugs can be delivered through the skin by CAP treatment. Two distinct deliveries may be of special interest for wound scaffolds: platelet-rich plasma (PRP) and stem cells. Besides electrospinning with prepared blend solutions containing PRP or stem cells, the scaffolds could be augmented by 3D printing technologies depositing PRP or stem cells into prepared scaffolds [25]. PRP is an orthobiologic substance of particular interest. PRP is obtained by centrifugation of a patient’s whole blood. The components of PRP enable the accelerated growth of various tissues with minimal side effects. The local release of growth factors and cytokines stimulates wound healing [118]. A recent systematic review revealed that PRP application can accelerate wound closure. However, it does not affect wound infection rates and graft take rate [119]. Stem cells, for example, from fat cells, have been utilized for wound treatment. Fat grafts contain adipose-derived stem cells (ADSC), but low survival of cells within the grafts is a major limitation. Nolan et al. [120] reviewed the literature on fat grafting together with PRP therapy. Their recent survey suggests that there is a benefit of ADSC, particularly in impaired wound healing. Umbilical cord blood-derived CD34+ stem cells added to a polyether-sulfone nanofibrous scaffold led to healing of diabetic skin wounds [121,122]. Fu et al. [97] added stem cells obtained from urine (USC) to a PCL-gelatin blended scaffold and found accelerated angiogenesis promoting wound healing. In general, 3D bioprinting of skin scaffolds is becoming more and more a reality. It can be viewed as a manufacturing platform to establish a scaffold layer by layer consisting of biomaterials, human cells of different origin (e.g., keratinocytes, fibroblasts, but also immune cells or melanocytes) and cytokines, chemokines and growth factors [123]. For example, Albanna et al. reported in 2018 a proof-of-concept study utilizing a mobile skin bioprinting system [124]. Dermal fibroblasts and epidermal keratinocytes from the same patient or an allogenic donor were delivered directly to a skin injury as a replacement of the layered skin structure. The authors reported quick closure of the skin defect and a short epithelialization phase in animal models.

## 5. Conclusions

Wound healing is a complex sequential process that is far from being fully understood on the molecular level. New insights into wound healing leading to new therapeutic approaches are urgently needed, as chronic wounds pose a high burden in elderly populations. CAP treatment can foster the healing process by acidification, angiogenesis, enhanced dermal blood flow, and cell stimulation. Since the last decade and the certification and use of plasma medical devices in 2013 onwards in Germany to treat leg ulcers, the safety of cold atmospheric plasma is undebatable and has been shown by several research groups and companies. Moreover, its efficacy in accelerating wound healing in vivo has been demonstrated in several prospective human trials. Still, open questions remain as to the frequency of plasma application: up to 90 seconds several times per day, once daily, every second day, or only once a week? These questions will be answered by larger observational trials that are ongoing. As CAP is applied in addition to modern wound care and available wound dressings, the idea is appealing to combine CAP with even more effective dressings/scaffolds to utilize the above-mentioned plasma wound healing effects together with advanced abilities of dressings for cell migration and proliferation. New bioengineered nanofibrous scaffolds mimicking the skin, or HHP-devitalized human skin, can be the backbone of a new generation of wound dressings. Here, challenges remain as to the safety and non-toxicity of scaffold material including the absence of allergic potential as well as environmental degradability. This has to be combined with sufficient mechanical strength of scaffolds and their properties to replace skin defects and guide different cell types into the skin defect. Finally, cost-effectiveness in production and use—ideally one application, degradation of the scaffold in weeks, and replacement of the degraded scaffold by skin—has to be considered. We put the vision forward to combine CAP treatment with such dressings to synergistically accelerate the healing process, especially if the scaffold is augmented with substances such as platelet rich plasma or stem cells.

## Data Availability

Not applicable.
